# Knowledge and attitudes of pediatric dentists regarding temporomandibular disorders

**DOI:** 10.22514/jofph.2025.052

**Published:** 2025-09-12

**Authors:** Irene Aurora Espinosa de Santillana, Gabriel Muñoz Quintana, Gisela Nataly Rubin de Celis Quintana, Olga Patricia López Soto, Adriana María Martínez Hernández, Guillermina Coba Mendoza

**Affiliations:** ^1^Faculty of Stomatology, Meritorious Autonomous University of Puebla, 72000 Puebla, PUE, Mexico; ^2^Department of Oral Health, Autonomous University of Manizales, 170001 Manizales, Colombia

**Keywords:** Temporomandibular disorders, Temporomandibular joint, Knowledge, Pediatric dentists, Children

## Abstract

**Background**: Temporomandibular disorders (TMD) encompass musculoskeletal and neuromuscular conditions affecting the temporomandibular joint (TMJ), masticatory muscles and other associated structures. While all dentists, regardless of their specialty, should possess a comprehensive understanding of TMD diagnosis and treatment, there is a limited number of studies assessing the knowledge and attitudes of pediatric dentists on this subject. The objective was to evaluate the knowledge and attitudes of pediatric dentists regarding the diagnosis and management of TMD in pediatric patients. **Methods**: This observational, analytical, cross-sectional study included 266 pediatric dentists who completed a 41-item questionnaire. Of these, 35 items assessed knowledge, while six evaluated attitudes. Descriptive statistics were analysed, and the chi-square test was applied for comparisons, with statistical significance set at *p* < 0.05. **Results**: The mean correct response rate across the four assessed domains was 49.5%. The lowest accuracy was observed in the pathophysiological domain (33%), followed by the psychophysiological domain (50%), the psychiatric disorders domain (64.1%) and the chronic pain domain (50.4%). **Conclusions**: Pediatric dentists exhibited limited knowledge of TMD but expressed positive attitudes toward its diagnosis and treatment. Morever, they demonstrated a lack of awareness regarding their role in preventive education on this condition.

## 1. Introduction

The American Academy of Pediatric Dentistry defines temporomandibular disorders (TMD) as a group of musculoskeletal and neuromuscular conditions characterized by a range of clinical signs and symptoms affecting the temporomandibular joint (TMJ), masticatory muscles and associated structures [1]. Although TMD is often associated with adults, it can also affect pediatric patients, with the common symptoms comprising myofascial pain, headaches, neck pain, difficulty chewing and clicking sounds during mouth opening and closing [2].

The prevalence of TMD has increased significantly in recent years, not only among adults but also in pediatric patients. According to the American Academy of Orofacial Pain, TMD-related signs and symptoms are less commonly reported as chronic complaints in pediatric patients. However, when present, they can significantly affect the muscles of mastication, the TMJ and associated structures.

Since 1993, multiple studies have reported a lack of knowledge regarding TMD among dental professionals across various specialties, including general dentistry, orthodontics and TMD specialists, and this knowledge gap is particularly pronounced among general practitioners. Tegelberg *et al*. [3] evaluated self-perceived knowledge, attitudes, and clinical experience in managing TMD among general dentists in Sweden, and their findings indicated that while dentists exhibited a positive attitude toward treating children and adolescents with TMD, many lacked established diagnostic protocols and standardized decision-making processes for treatment.

Taqi *et al*. [4] investigated the knowledge and beliefs of general dentists in Korea regarding TMD. Their findings revealed deficiencies in the pathophysiological aspects. Similarly, Prabhakar *et al*. [5] reported in 2024 that TMD general dentists possessed significantly greater knowledge than specialists. Furthermore, their study suggested that attitudes toward TMD became less favorable with increasing age and years of clinical practice.

Espinosa *et al*. [6] conducted a cross-sectional observational study involving 161 dental educators in Puebla, Mexico, and reported significant inconsistencies in participants’ knowledge of TMD, highlighting the low priority given to this field in dental education and the substantial variability in diagnostic and treatment expertise, particularly regarding pathophysiology. Similarly, Cintra *et al*. [7] conducted a comparative analysis of TMD knowledge and beliefs among specialists in TMD and Orofacial Pain using the same survey instrument employed two decades earlier, and despite the inclusion of new questions addressing pathophysiology and chronic pain, no significant improvements in knowledge were observed. Moreover, Al-Huraishi *et al*. [8] assessed TMD knowledge among recent dental graduates and specialists and found that recent graduates had limited knowledge across all assessed domains compared to specialists, underscoring a major deficiency in dental education curricula. Mozhdeh *et al*. [9] evaluated TMD knowledge among general dentists and specialists in Italy and their findings demonstrated that only half of participants had proper knowledge (41% acceptable and 12% good) regarding TMD.

In 2023, Tormes *et al*. [10] evaluated predoctoral dental students in northeastern regions and observed an overemphasis on occlusal factors and a lack of confidence in managing TMD. Similarly, Xiong *et al*. [11] assessed the knowledge and attitudes of general dentists and postgraduate students, finding that both groups demonstrated inadequate knowledge on TMD management. These findings highlight persistent deficiencies in TMD-related education and training among dental professionals.

Despite the increasing recognition of TMD in clinical practice, considerable variability remains in the knowledge and training that dentists receive regarding its diagnosis and treatment [10]. While several studies have explored TMD awareness among general dentists and specialists, limited research has focused on pediatric dentists [11]. Given that pediatric dentists often serve as the first point of contact for children, assessing their understanding of TMD is essential for ensuring early diagnosis and appropriate management. A comprehensive evaluation of their knowledge and attitudes may provide valuable insights for developing targeted educational strategies to improve competency in this field. Therefore, this study aimed to assess the knowledge of pediatric dentists regarding TMD and their attitudes toward diagnosing and treating pediatric patients with this condition.

Further emphasizing the significance of this issue, Minervini *et al*. [12] conducted a systematic review in 2023 to summarise evidence on TMD prevalence in pediatric populations. Their findings indicated that TMD is more prevalent in females than in males, with reported prevalence rates ranging from 20% to 60% among children. Given the high prevalence of TMD and its potential impact on oral function and overall well-being, early identification and intervention are crucial in preventing disease progression.

As primary care providers for pediatric patients, pediatric dentists are in a unique position to recognize early signs of TMD, provide timely interventions and educate caregivers about preventive strategies. Strengthening their knowledge and clinical expertise in this area is, therefore, essential to reducing the risk of untreated TMD evolving into more complex conditions that may compromise quality of life.

## 2. Materials and methods

### 2.1 Study design

A cross-sectional, observational, and analytical study was conducted to evaluate the knowledge and attitudes of pediatric dentists affiliated with the Mexican Association of Pediatric Dentistry (AMOP) regarding TMD. A total of 266 pediatric dentists voluntarily participated and completed a 41-item questionnaire, which included 35 questions assessing knowledge and six questions evaluating attitudes. The estimated response time for the questionnaire was 10 minutes.

To assess knowledge, the study employed an instrument originally proposed by Linda Le Resche in 1993 [13]. The questionnaire was reviewed for its validity by two pediatric dentists with over 20 years of experience in diagnosing TMD. The items were categorized into four domains: Pathophysiological, Psychophysiological, Psychiatric Disorders and Chronic Pain.

Data collection was conducted during AMOP’s Magno Course in October 2022, where the questionnaire was made accessible via a QR code displayed at various strategic locations within the event venue and distributed through email. The study population comprised AMOP-affiliated pediatric dentists, including graduates with a specialization, master’s or doctoral degree in pediatric dentistry, as well as students from various universities in Mexico. Participation was entirely voluntary, and no financial incentives were provided.

As of 2022, AMOP had registered a total of 770 pediatric dentists, all of whom were invited to participate in the study. A total of 266 members agreed to participate, resulting in a response rate of 34.5%, which included 220 practicing pediatric dentists and 46 pediatric dentistry students. The inclusion criteria encompassed all AMOP-affiliated pediatric dentists who voluntarily agreed to participate. The exclusion criteria consisted of incomplete questionnaire responses. The study was approved by the Faculty of Stomatology Research Committee at Benemérita Universidad Autónoma de Puebla (BUAP) in July 2022 (approval no.: 2022179). The study adhered to the Mexican health research regulations and complied with the ethical principles outlined in the Declaration of Helsinki (2025) [14].

### 2.2 Questionnaire

The questionnaire was designed to collect general demographic and professional information, including the respondent’s email address, gender, university of graduation, level of education and year of graduation. Following this, the original instrument developed by Le Resche was used to assess both knowledge and attitudes related to TMD.

Knowledge-based responses were recorded on a three-point Likert scale with the options: “Agree”, “Disagree”, and “Prefer not to answer”. A response was considered correct if it aligned with the consensus of 13 American specialists in TMD and Orofacial Pain. Conversely, a response was classified as incorrect if it did not align with the consensus or if the respondent selected the “Prefer not to answer” option. The attitude component of the questionnaire consisted of six items, each requiring a dichotomous response (“Yes” or “No”).

### 2.3 Statistical analysis

All categorical variables were summarized using frequencies and percentages, while continuousvariables were presented using measures of central tendency and dispersion. A chi-square test was applied to compare TMD knowledge between AMOP-affiliated pediatric dentists and specialists in TMD and Orofacial Pain, with statistical significance set at *p* < 0.05

## 3. Results

Of the 770 AMOP-affiliated pediatric dentists, 266 participants responded to the survey, yielding a response rate of 34.5%. Among the respondents, 182 were women (68.4%) and 84 were men (31.6%). The participants represented 66 institutions, including 25 public universities (37.8%) and 41 private universities (62.2%). Of the 25 public universities, 24 were represented by 198 respondents (74.4%), while 41 private universities accounted for 68 respondents (25.6%).

All respondents had obtained educational qualifications beyond a bachelor’s degree, including specialization, master’s or doctoral degrees. Among them, 220 participants (82.7%) were practicing pediatric dentists, while 46 (17.3%) were pediatric dentistry students. The mean number of years since graduation was greater than nine, with a median of six years (Table 1).

**Table 1.** Demographic characteristics of study participants.

Demographic Characteristics		Frequency, n (%)
Gender		
	Men	84 (31.6%)
	Women	182 (68.4%)
University		
	Public	198 (74.4%)
	Private	68 (25.6%)
Education Level		
	Master’s (Graduates)	220 (82.7%)
	Bachelor’s (Students)	46 (17.3%)
Years Since Graduation		
	Less than 10	184 (69.2%)
	11 to 20	46 (17.3%)
	21 to 30	21 (7.9%)
	31 to 40	13 (4.8%)
	More than 41	2 (0.8%)

The overall correct response rate across the four assessed domains was 49.5%. The pathophysiological domain had the lowest accuracy, with a correct response rate of 33.7% (Table 2). A notable misconception was observed regarding occlusal interferences, a concept evaluated in the pathophysiological domain. While occlusal interferences have traditionally been considered a risk factor for TMD development, this belief is not supported by current evidence. However, when assessing this concept, the majority of surveyed pediatric dentists incorrectly associated occlusal interferences with TMD, with only 9.8% providing a correct response.

**Table 2.** Comparison of results according to specialists in TMD in the pathophysiological domain.

Pathophysiological Domain	Specialists in TMD and Orofacial Pain	Pediatric Dentists	Chi-square *p*-value
Balancing interferences are commonly related to TMD (Disagree).	85.0%	9.8%	0.01
Occlusal equilibration is a useful early treatment for TMD (Disagree).	85.0%	3.8%	<0.001
Orthodontic or orthodontic treatment can prevent the onset of TMD (Disagree).	77.0%	10.5%	0.01
Arthroscopic surgery is almost completely effective in repositioning the disc in patients with internal derangements (Disagree).	100.0%	30.1%	0.31
Orthodontic therapy is the best treatment to resolve TMD in a patient with skeletal malocclusion (Disagree).	92.0%	60.2%	0.71
TMD caused by trauma is much more difficult to treat and has a far worse prognosis than other types of TMD (Disagree).	83.0%	36.5%	0.42
Transcranial films are the most accurate method for viewing the TM Joint (Disagree).	77.0%	43.6%	0.52
The presence of arthritic changes on tomograms, along with crepitus in the joint, indicates the need for treatment.	77.0%	10.9%	0.01
The position of the condyle in the fossa, as seen in tomograms, is a very accurate indication of internal derangement (Disagree).	92.0%	32.0%	0.34
Mandibular reposition splints are more effective than maxillary splints (Disagree).	100.0%	27.8%	0.27
Nocturnal bruxism is caused by occlusal interferences (Disagree).	85.0%	65.4%	0.76
Ice packs and/or heat packs and passive muscle stretching are good early treatments for TMD (Agree).	100.0%	66.9%	0.78
All individuals with clicking TMJs require treatment (Disagree).	100.0%	41.0%	0.48
Median percentage of right answers according to specialists in TMD: 33.7%			

*TMD: Temporomandibular disorders; TMJ: temporomandibular joint.*

Similarly, only 3.8% of respondents disagreed with the statement that occlusal balance is a useful treatment for TMD, suggesting that more than 96% of pediatric dentists may be implementing ineffective treatments.

Additionally, when asked whether orthopedic or orthodontic treatments could prevent the onset of TMD, nearly 90% of respondents incorrectly believed this to be true. However, findings from specialists in TMD and Orofacial Pain, as well as existing literature, indicate that these treatments do not prevent TMD development.

Furthermore, nearly 90% of respondents agreed that the presence of arthritic changes on tomographic imaging, along with joint crepitus, necessitates treatment. However, this perspective contradicts the consensus among TMD and Orofacial Pain specialists and the evidence available in the literature.

In the psychophysiological domain, the overall correct response rate was 50% (Table 3). One of the assessed items examined whether tension and stress increase electromyographic activity in the mandibular muscles of susceptible patients. While most TMD and Orofacial Pain specialists agree with this statement, only 3% of surveyed pediatric dentists provided responses aligning with the specialists’ consensus.

**Table 3.** Comparison of results according to specialists in TMD in the psychophysiological domain.

Psychophysiological Domain	Specialists in TMD and Orofacial Pain	Pediatric Dentists	Chi-square *p*-value
The mechanisms of acute and chronic pain are the same (Disagree).	100.0%	87.2%	0.92
Biofeedback can be useful for treating TMD (Agree).	100.0%	40.6%	0.48
Oral parafunctional habits are often significant in the development of TMD (Agree).	85.0%	86.5%	0.92
Patients with TMD who clench/brux do so either during the day or at night, but not both (Agree).	85.0%	18.4%	0.11
Stress management is indicated for many TMD patients (Agree).	77.0%	95.1%	0.97
Stress is a major factor in the development of TMD (Agree).	100.0%	92.9%	0.96
Tension and stress increase jaw muscle EMG levels in susceptible patients (Agree).	82.0%	3.0%	<0.001
Progressive muscle relaxation is not an effective treatment for TMD (Agree).	92.0%	23.7%	0.20
Information on the daily pattern of TMD symptoms can help identify contributing factors (Disagree).	92.0%	26.0%	<0.001
Median percentage of right answers according to specialists in TMD: 50%			

*TMD: Temporomandibular disorders.*

The psychiatric disorders domain demonstrated an average correct response rate of 64.1% (Table 4), while the chronic pain domain exhibited an average of 50.4% (Table 5). In these two domains, no significant differences were observed between TMD and Orofacial Pain specialists and pediatric dentists in terms of knowledge accuracy.

**Table 4.** Comparison of results according to specialists in TMD in the psychiatric disorders domain.

Psychiatric Disorders Domain	Specialists in TMD and Orofacial Pain	Pediatric Dentists	Chi-square *p*-value
Clinical depression is rare in chronic TMD patients (Disagree).	100.0%	49.2%	0.59
Depressed mood is fairly common in chronic TMD patients (Agree).	86.0%	64.7%	0.76
Anxiety disorders are more common in TMD patients than in the population at large (Agree).	79.0%	71.4%	0.81
Depression can be an important etiologic factor in chronic pain (Agree).	79.0%	71.1%	0.81
Median percentage of right answers according to specialists in TMD and Orofacial Pain: 64.1%			

*TMD: Temporomandibular disorders.*

**Table 5.** Comparison of results according to specialists in TMD in the chronic pain domain.

Chronic Pain Domain	Specialists in TMD and Orofacial Pain	Pediatric Dentists	Chi-square *p*-value
Chronic TMD patients should be advised to rest and limit their work and social activities when they are experiencing pain (Disagree).	85.0%	42.9%	0.51
PRN narcotics (*i.e.*, “as needed” for pain) are a treatment of choice when TMD pain is severe (Disagree).	93.0%	19.2%	0.12
Antidepressants are never indicated in the management of TMD (Disagree).	88.0%	43.2%	0.52
An extensive history of previous treatment failures in a TMD patient is usually an indication for surgery (Disagree).	100.0%	35.0%	0.39
Chronic pain is a behavioral as well as a physical problem (Agree).	96.0%	66.5%	0.77
Although some TMD patients have psychological problems, these problems are usually unrelated to their pain (Disagree).	85.0%	38.3%	0.45
Difficulty with sleep is a common finding in chronic pain (Agree).	96.0%	78.9%	0.87
Some patients use pain as an excuse to avoid unpleasant chores (Agree).	89.0%	54.5%	0.66
Behavior modification treatments are appropriate for patients with chronic TMD pain (Agree).	88.0%	75.2%	0.84
Median percentage of right answers according to specialists in TMD: 50.4%			

*TMD: Temporomandibular disorders; PRN: “as needed”.*

Attitudes of Pediatric Dentists Toward TMD.

The survey results indicated that pediatric dentists exhibited a favorable attitude toward diagnosing and managing TMD. Among the six attitude-related questions, 83.1% of respondents expressed a willingness to provide information, care, diagnosis, and treatment for pediatric patients with TMD. However, 16.9% of the respondents showed a median negative attitude regarding the items considered in this category (Table 6).

**Table 6.** Attitudes of Pediatric Dentists.

Items	Negative	Positive
Pediatric dentists should be the main provider of information and advice on TMD	31.2%	68.8%
Pediatric dentists should examine and diagnose TMD during routine wellness checkups	3.0%	97.0%
Would refer a patient with TMD to another specialist?	19.9%	80.1%
Would examine the TMJ of patients for signs and symptoms of TMD?	3.0%	97.0%
Would provide advice on TMD prevention to parents of patients?	5.7%	94.3%
As part of their standard practice, they conduct preventive consultations for TMD.	38.4%	61.6%
Median percentage of attitudes answers	16.9%	83.1%

*TMD: Temporomandibular disorders.*

When asked whether pediatric dentists should assume the role of providing information and guidance on TMD, 31.2% of respondents stated that they did not consider this to be part of their professional responsibilities. Nevertheless, 94.3% of respondents reported that they would provide advice or recommendations to parents regarding TMD prevention.

## 4. Discussion

This study assessed the knowledge and attitudes of 266 pediatric AMOP dentists regarding TMD. The sample size is comparable to that of previous studies conducted in different countries using the same assessment instrument, thereby allowing for meaningful comparisons.

The results demonstrated that pediatric dentists exhibited limited knowledge of TMD, with an overall correct response rate of 49.5% across the four assessed domains. This finding aligns with the results reported by Le Resche *et al*. [13] who evaluated 386 general and specialist dentists and observed similar deficiencies in TMD-related knowledge. The persistence of these gaps over time suggests that the etiology, diagnosis, and treatment of TMD remain poorly understood, particularly within the pathophysiological and psychophysiological domains.

Among the four domains, the pathophysiological domain had the lowest correct response rate (33.1%), indicating a widespread misunderstanding of the biological mechanisms underlying TMD. This result is consistent with previous findings by Taqi *et al*. [4] and Al-Huraishi *et al*. [8] (2020), who also identified the pathophysiological domain as the area with the most pronounced knowledge deficiencies among general dentists. Similarly, Tormes *et al*. [10] (2023) reported significant gaps in practitioners’ understanding of the relationship between occlusion and TMD, further highlighting the persistent misconceptions in this area. Additionally, Xiong *et al*. [11] (2023) found that many dentists lacked adequate training, understanding, and confidence in TMD management, reinforcing the need for improved educational strategies.

A key factor contributing to the low correct response rate in the pathophysiological domain may be the misconception among pediatric dentists that their knowledge of TMD is sufficient despite clear evidence to the contrary. The variability in knowledge may also be linked to differences in educational methodologies at the university level. Studies have shown that curricula incorporating extensive clinical training are more effective in enhancing knowledge acquisition than traditional lecture-based approaches [8].

Misconceptions regarding the role of orthodontic and orthopedic treatments in TMD prevention were also evident in this study. When asked whether these treatments could prevent the onset of TMD, nearly 90% of respondents incorrectly believed that they could, while only 10.5% provided responses aligning with expert consensus. The association between orthodontic treatment and TMD remains a controversial topic in the scientific community. However, a systematic review by Fernández-González *et al*. [15] (2015) found no evidence supporting a causal relationship between orthodontic treatment and TMD. Furthermore, the review was unable to determine whether such treatments could prevent or improve TMD, emphasizing the need for caution when making clinical recommendations in this area.

Another widely held misconception among respondents was the belief that arthritic changes observed on tomographic imaging, in conjunction with joint crepitus, necessitate treatment. Nearly 90% of participants considered these findings to be definitive indicators for intervention, a perspective that contradicts both expert consensus and existing literature. While crepitus is commonly associated with degenerative joint disease of the TMJ, it does not necessarily indicate the need for treatment [16].

The survey assessing pediatric dentists’ attitudes toward TMD revealed that while most respondents were willing to diagnose and treat TMD, a significant proportion (30%) did not consider preventive education on TMD to be within their professional responsibilities. This suggests that many pediatric dentists may view their role as limited to managing traditional dental issues rather than extending to more complex conditions such as TMD, which often require a multidisciplinary approach. Such perceptions may reduce motivation to provide early education and preventive guidance, which is essential for minimizing the progression and long-term impact of TMD [17].

The findings of this study underscore the need to enhance TMD-related education in pediatric dentistry training programs. Addressing misconceptions regarding etiology, diagnosis, and management strategies may help improve clinical decision-making and ultimately enhance patient outcomes.

Most pediatric dentists (55%) exhibited a favorable disposition toward addressing TMD in children and adolescents. However, they demonstrated limited awareness of their role in preventive education for parents and children, which may be attributed to deficiencies in their ability to accurately diagnose and treat TMD, as well as a lack of confidence in the effectiveness of preventive education and non-therapeutic management strategies.

Since 2001, multiple studies, including those by Tegelberg *et al*. [3] (2016), Xiong *et al*. [11] (2023), and Liu *et al*. [17] (2024) have emphasized the need for specialized training in TMD and the development of standardized referral protocols. The findings of the present study further highlight this issue, as only 19.9% of respondents reported having referred patients for specialist consultation. This suggests that a considerable proportion of pediatric dentists may not fully recognize the severity or complexity of TMD in pediatric patients, potentially leading to an underestimation of the need for specialist referrals. Given that TMD cases in children are often perceived as mild or self-limiting [18], many pediatric dentists may opt for observation rather than referral.

More recently, in 2023, Razdan *et al*. [19] conducted a questionnaire-based study to assess knowledge and awareness of TMD among Indian dentists. Similar to the present study, their findings reflected that the knowledge of physiological anatomy along with the management of symptomatic TMD is insufficient.

## 5. Limitations

The study had several limitations worth highlighting. First, since the respondents were exclusively AMOP-affiliated pediatric dentists, the data cannot be generalized to other populations. Nevertheless, the utilized instrument is the most employed globally, facilitating the comparison of findings with those documented in existing literature. Another limitation is that a substantial proportion of respondents were postgraduate students and recently graduated pediatric dentists. Specifically, 40% of participants had less than five years of professional experience, which may have influenced their responses.

## 6. Conclusions

The findings of this study indicate that pediatric dentists affiliated with AMOP have a limited understanding of TMD compared to specialists in TMD and Orofacial Pain. Despite their positive disposition toward diagnosing and treating TMD, pediatric dentists often overlook their role in preventive education. This suggests that many pediatric dentistry specialists may lack the necessary knowledge and clinical proficiency to accurately diagnose and provide effective treatment for pediatric patients with TMD.

The underdiagnosis of TMD in children and adolescents may have significant implications, as untreated conditions could lead to chronic dysfunction affecting oral health and quality of life in adulthood. Given these findings, undergraduate and postgraduate dental programs should critically evaluate and refine their curricula to ensure that future professionals receive comprehensive training in TMD diagnosis, management, and prevention.

While the existing literature provides extensive knowledge on TMD, this study highlights the need for a stronger integration of theoretical knowledge with clinical practice. Thus, developing curricular improvements that enhance both conceptual understanding and hands-on experience could help bridge this knowledge gap. In addition, future research could focus on assessing the impact of such educational interventions on pediatric dentists’ competency in TMD management and tracking knowledge progression over time.
